# Future directions for notifiable diseases: tuberculosis-related laws in the Philippines

**DOI:** 10.1186/s12992-018-0405-2

**Published:** 2018-08-22

**Authors:** Yuri Lee

**Affiliations:** Graduate School of Public Health, Yonsei University Health System, Room 410, Administration B/D, 50-1 Yonsei-Ro, Seodaemun-Gu, Seoul, 120-752 Republic of Korea

**Keywords:** Notifiable disease, Tuberculosis, TB, Legislation, Regulation, Philippines

## Abstract

**Background:**

With the increasing burden of tuberculosis (TB) in the Philippines, and the risk of multidrug resistance to TB, there is a need to strengthen the surveillance system. In many countries, cases of TB are reported to health authorities, and reporting is an effective way to manage TB. Although TB is a universal and representative reportable disease, the Philippines does not designate it as a notifiable disease.

**Main text:**

This study aimed to review and compare current communicable disease-related laws and regulations in the Philippines with relevant international laws and regulations in other countries, to highlight where current TB notification regulations require change, or to determine whether they reflect global trends. Furthermore, we aimed to have TB included along with other communicable diseases on the list of legally required notifiable diseases in the Philippines.

We reviewed current TB-related laws, acts of parliament, executive orders, presidential decrees, administrative orders, and memorandums. We undertook a literature review of relevant World Health Organization documentation, with 17 countries selected for comparison. Data on reported TB cases in the Philippines were obtained from health authorities, and health legislation data from foreign countries was collected from a public law database or from the government websites of each country.

Most of the selected countries have a legislative basis for regulating notifiable diseases. In many countries, including Australia, Canada, China, Kiribati, Nauru, Niue, New Zealand, the Republic of Korea, Singapore, the United Kingdom, and Vanuatu, laws on communicable disease notification include TB notification. Our results suggest that notification of communicable diseases should be enforced through domestic health legislation.

**Conclusion:**

To align the Philippines with standard practice in the selected countries, TB could be included on the list of notifiable diseases in one of two ways. First, the current regulation “Revised List of Notifiable Diseases, Syndromes, Health-related Events and Conditions of 2008” could be revised to include TB. Second, new TB regulations could be introduced and implemented. Any revisions or new regulations should specify methods to identify and manage TB, and safeguard individual rights.

## Background

Tuberculosis (TB), a serious communicable disease, remains a significant public health threat worldwide [8, 12]. In many countries, it is a requirement that TB cases be reported to health authorities to facilitate monitoring the disease incidence and managing this infectious disease effectively. Although TB is a universal and representative reportable disease, the Philippines does not designate TB as a notifiable disease. Generally, TB is a communicable disease notifiable by law because it has the potential to become a significant epidemic with high transmission rates in some foci [10]. Moreover, there may be a rapid emergence of multidrug-resistant TB (MDR-TB) and extensively drug-resistant TB (XDR-TB) hot spots [11].

**Table 1:** Comparison of Laws on Notifiable Diseases (specifically, Tuberculosis) Between the Philippines and Other Countries

Country	Existence of Law	Name of Law (Only Primary and Subsidiary Legislations)	Notifiable Diseases by Law	Note
Australia	Yes	-National Health Security Act, No. 174, 2007 (Primary Legislation) -National Notifiable Diseases List, 2008 (Subsidiary Legislation, under the Prime Minister)	In 2010, 65 diseases and conditions were notifiable in Australia by law. -Bloodborne Disease –Gastrointestinal –Quarantinable -Sexually transmissible -Vaccine preventable –Vectorborne –Zoonoses -Other bacterial infections (including Tuberculosis)	Annual report has been published on the website of Australia’s Department of Health and Ageing http://www.health.gov.au/internet/main/publishing.nsf/content/cda-pubs-annlrpt-nndssar.htm
Canada	Yes	The list of nationally notifiable diseases was revised and published in 2008. *(Erratum: Final report and recommendations from the National Notifiable Diseases Working Group*. CCDR 2008;34:24–5)	-Enteric, food, and waterborne diseases -Diseases transmitted by respiratory routes -Diseases transmitted by direct contact and through the provision of health care -Disease preventable by routine vaccination -Sexually transmitted and blood-borne pathogens -Vector-borne and other zoonotic diseases (including TB) -Worldwide potential bioterrorism agents	Public Health Agency in Canada http://diseases.canada.ca/notifiable/diseases-list
China	Yes	-Law on the Prevention and Treatment of Infectious Disease	Law requires mandatory reporting for many infectious diseases. -Class A includes plague and cholera. -Class B includes 25 diseases, such as viral hepatitis. -Class C includes 10 diseases, such as influenza. -HIV/AIDS, gonorrhea, and syphilis were added in 1990and TB was added in 2004.	http://csis.org/files/media/csis/pubs/090325_freeman_chinacapacity_web.pdf
Cook Islands	Diseases not specified	-Public Health Act 2004	Part 11 states the notifiable and dangerous conditions.	http://www.paclii.org/ck/legis/num_act/pha2004126/
Fiji	Diseases not specified	-Public Health Act	Part 7. Infectious Disease Section 71 Notification of the Infectious disease includes the notification requirements described under the Public Health Act.	http://www.paclii.org/fj/legis/consol_act_OK/pha126/
Kiribati	Yes	-The Public Health Ordinance [Cap 80] and Public Health Declarations	Section 21 states that every case of infectious disease is to be reported to the nearest sanitary inspector.	http://www.paclii.org/ki/legis/consol_act/pho179/
Lao People’s Democratic Republic	Diseases not specified	-Law on Hygiene, Disease Prevention and Health Promotion 2001	Chapter 3. Article 26 mentions the communicable diseases.	http://www.vientianetimes.org.la/
Malaysia	Diseases not specified	-Prevention and Control of Infectious Diseases Act 1988 Amended 1997	Part IV Control of the Spread of Infectious Disease Section 10. Requirement to report infectious disease	http://www.legalinfo.mn/
Nauru	Yes	- Diseases Ordinance 1923 - The Tuberculosis Ordinance 1967	The Tuberculosis Ordinance 1967 contains specific examination, treatment, and notification requirements in relation to tuberculosis.	http://ronlaw.gov.nr/nauru_lpms/files/acts/5db193437d21ff3bcd289d872282a2ae.pdf
Niue	Yes	-Public Health Act 1965	Chapter 4 describes the notifiable infectious diseases, including tuberculosis, leprosy, venereal diseases, including syphilis, gonorrhea, and soft sore, and any other infectious disease, which the Cabinet may declare by publishing a public notice.	http://www.paclii.org/
New Zealand	Yes	-Health Act 1956 -Tuberculosis Act 1948	-Section A. Infectious Diseases Notifiable to a Medical Officer of Health and Local Authority (*n* = 12) -Section B. Infectious Disease Notifiable to Medical Officer of Health (*n* = 33) -Tuberculosis (all forms)	https://www.health.govt.nz/our-work/diseases-and-conditions/notifiable-diseases/
Philippines	Yes, but TB is not included.	-The Law of Reporting of Communicable Disease (Republic Act 3573) -Department Circular No. 176 series of 2001 -Revised List of Notifiable or Reportable Diseases 2008 (Administrative Order No. 2008–0009)	-Category 1. Immediately Notifiable Disease/Syndrome/Events and Conditions (*n* = 13) -Category 2.Weekly Notifiable Diseases or Syndromes (*n* = 14)	Revised List of Notifiable or Reportable Diseases 2008
Republic of Korea	Yes	-Infectious Disease Control and Prevention Act, -Quarantine Act	-Group 1. Infectious Disease (drinking water or foodborne) -Group 2. Infectious Disease (Vaccine preventable) -Group 3. Infectious Disease (requiring continuous surveillance and establishment of control measures, including TB) -Group 4. Infectious Disease (newly broken out or overseas epidemic) -Group 5. Infectious Disease (parasite infection)	https://elaw.klri.re.kr/kor_service/lawView.do?hseq=40184&lang=ENG
Samoa	Diseases not specified	-The Health Ordinance 1959	-Article 31. Registry of Infectious Diseases	Ministry of Health posts schedule of notifiable diseases (last updated April 2013) http://www.paclii.org/ws/legis/consol_act/ho1959122/
Singapore	Yes	-Infectious Disease Act 1976	Section 6. Notification of Infectious Disease (total *n* = 37) −24 h from time of diagnosis to director, Communicable Disease Division, MOH (*n* = 19) − 72 h from time of diagnosis to director Communicable Disease Division, MOH (*n* = 9) − 72 h from time of diagnosis to head, National Public Health Unit, MOH (*n* = 2) − 72 h from time of diagnosis to director, Tuberculosis Control Unit, STEP Registry (*n* = 1) − 72 h from time of diagnosis to head, Department of STI Control Clinic (*n* = 5) − 72 h from time of diagnosis to director, National Skin Centre, Leprosy Registry (*n* = 1)	https://www.moh.gov.sg/content/moh_web/home/diseases_and_conditions.html
Tonga	Diseases not specified	-Public Health Act 2008	It creates notification requirements and public health responses for certain communicable diseases (“notifiable conditions”).	http://www.paclii.org/to/legis/num_act/pha2012206/
United Kingdom	Yes	-Public Health (Control of Disease) Act 1984 -Public Health (Infectious Disease) Regulations 1998	The new legislation adopts an all hazards approach. In addition to the specified list of infectious diseases, there is a requirement to notify cases of other infections or contamination, which could present a significant risk to human health. List of Notifiable Diseases (*n* = 32 including Tuberculosis)	https://www.gov.uk/government/publications/notifiable-diseases-weekly-reports-for-2018
Vanuatu	Yes	-The Public Health Act	Part 3. Prevention and Suppression of Notifiable Disease (Article 8. Reporting of Notifiable Disease–Article 21)	http://www.paclii.org/

Legal preparedness and key regulations are central in ensuring the success of public health efforts to control the spread of TB [13]. One of the epidemiological indicators for national TB control programs is the case detection rate for all forms of TB [2, 16].

However, TB has not been included on the list of legally notifiable diseases in the Philippines since 2001 when Department Circular No. 176 was revised. This has limited the ability of the Department of Health (DOH) to ensure notification from non-national TB providers in the public and private sectors to monitor the disease incidence effectively in the country [9, 15, 18]. This is a major concern, as the incidence of TB cases cannot be estimated accurately due to the inadequate reporting of TB cases from non-NTP providers in the public and private sectors.

This study aimed to review and describe TB regulations in the Philippines to assist public health practitioners and policy makers to assess and improve regulations, as a tool to control TB. Regulating TB as a notifiable disease improves the detection and surveillance of the disease [3]. This study also aimed to provide options for introducing TB notification regulations. The current communicable disease-related laws and regulations in the Philippines and those of other countries were compared to determine the comparable status of TB laws in the Philippines, and to show where the current TB regulations in the Philippines need to change to reflect global practice. Laws regulating TB are defined as comprising health legislation (e.g., acts and decrees) that directly mention “TB” or “notifiable disease” in the body of the law, with the main purpose limited to the control of TB.

## Methods

A review and a comparison of the current communicable disease-related laws and regulations in the Philippines with relevant international health laws and regulations and countries’ cases was conducted.

To review the current TB-related laws in the Philippines, relevant acts, executive orders, presidential decrees, administrative orders, and memorandums were reviewed. Global and regional documents from the World Health Organization (WHO) were also reviewed. Comparable health policies and legislations of countries in the Western Pacific region, in particular, as well as those of a range of other countries were reviewed. These selected countries included Australia, Canada, China, the Cook Islands, Fiji, Kiribati, Lao People’s Democratic Republic, Malaysia, Nauru, Niue, New Zealand, the Republic of Korea, Samoa, Singapore, Tonga, the United Kingdom, and Vanuatu for comparison, as relevant website data was available. The WHO have categorized the Philippines as one of the countries in the Western Pacific region; therefore, most countries within the region were selected for comparison, with developed countries such as the United Kingdom and Canada also selected for review. It was considered that the range of countries selected was sufficiently representative of TB regulations internationally to be informative of best policy and practice.

Data on the cases reported in the Philippines were obtained from health authorities, and health legislation data of foreign countries were collected from public law databases or from the webpages of each government (Table 1) that were publicly accessible. A content analysis of the legal provisions was performed to propose future directions of TB control law in the Philippines.

## Results

### The current situation in the Philippines

The Philippines is one of 22 countries with the highest TB burden that collectively account for more than 80% of reported TB worldwide. The Philippines has a population of 99 million and has a high burden of TB, with an estimated 290,000 new TB cases each year [17]. The estimated incidence of newly diagnosed TB annually was 324 per 100,000 persons in 2015 [22], and TB-related mortality was 33 per 100,000 persons in 2010 [15].

TB continues to be the sixth leading cause of death despite the long-standing public health prevention efforts made by various units of the Philippine government. The Philippines ranks fifth among the 27 priority countries with a high burden of multidrug-resistant TB (MDR-TB) [6], and MDR-TB has become a serious public health problem [5, 6] Currently, the number of patients enrolled for treatment only includes 25% of the estimated 12,000 MDR-TB cases [17].

A public health service package supported by the government is outlined by PhilHealth, a national health insurance scheme of the Philippines that covers 38% of the population. Services covered are not comprehensive, co-payment is high, and the reimbursement procedure is complex. Private services are used by approximately 30% of the population who can afford fee-for-service payment. The TB prevention program is operated by health centers and provincial hospital outpatient clinics in the public sector. In the private sector, there are 1090 TB-DOTS clinics, and those working in private hospitals, pulmonary specialists, and some general practitioners participate in the DOTS program. Outpatient TB-DOTS is included in the benefits package, but outpatient consultation and ongoing requirements for medication are not yet included [23]. Cost may be among the barriers in accessing TB services, although the national TB budget was 104 million US dollars in 2016 [21]. Regarding treatment outcomes and prevention, the TB treatment coverage in 2015 involved 85% of the reported and estimated incidence cases. Approximately 92% of new and relapsed cases registered in 2014 had been previously treated cases, excluding those relapsed cases (83%) registered in 2014 [22].

The use of NTP surveillance system data to report the number of TB cases in the Philippines has several limitations. Cases diagnosed and treated in health facilities outside the NTP network of providers, including private clinics and hospitals, are not included; therefore, the surveillance system underreports the total number of TB cases in the Philippines [18].

The Code of Sanitation of the Philippines (Presidential Decree No. 856) was instituted in 1975. In 1929, the Law on Reporting of Communicable Diseases was enacted, which required all individuals and health facilities to report notifiable diseases to local and national health authorities. In addition, the Act reorganized the TB Unit in the Department of Health to specify that the TB Unit have the functions of pooling all information on TB and exchanging such information with other countries. Section “Results” of the Law of Reporting of Communicable Disease states that a case of reportable or communicable disease shall include any person sick from, affected by, or attacked by a communicable disease; and that TB should be on the list of the reportable diseases.

The Law on Reporting of Communicable Diseases (Republic Act 3573) still requires all individuals and health facilities to report notifiable diseases to local and national health authorities. In 1990, the Department of Health circular No. 157 was issued, which comprised a list of reported diseases included on the reviewed list of notifiable or reportable diseases. In that circular, TB is listed as a reportable disease along with the case definitions of TB, meningitis, respiratory illnesses, and other types of communicable diseases. The 1997 revised list of notifiable diseases is the same as that of the 1990 document. However, the 2001 revised list of notifiable or reportable diseases included diseases and syndromes that had been selected because they were epidemic-prone diseases, targeted for eradication or elimination, and subject to international health regulations. TB was no longer included the list of reportable or notifiable diseases.

A recent update on the administrative Order no. 9 series (2008) includes a revised list of notifiable diseases, syndromes, and health-related events and conditions. It describes two categories of notifiable diseases: an “immediate” notifiable disease category consisting of 13 diseases and a “weekly” notifiable disease category, which includes 14 diseases. However, TB is not included in either of these categories.

### The need for designating TB as a notifiable disease according to global norms

The following international standards on notifiable disease reporting include: International Health Regulations [19], the Asia Pacific Strategy for Emerging Diseases [20], and the International Digest of Health Legislation (IDHL). The WHO has used the IDHL as a guide for domestic law since 1948. The IDHL includes norms and standards of legislation on communicable disease issues, comprising epidemic control measures, immunization and vaccination, notification requirements, prevention and control measures, and tests dealing specifically with HIV/AIDS. The Code of Sanitation of the Philippines notes that the Philippines recognized the [19] and that some of the provisions in the IHR may be considered part of this code.

The reporting of TB as a notifiable disease is intended to control TB through ensuring that healthcare professionals have an official obligation to inform the government concerning TB to enable it to control the disease and authorize health officials to act as necessary. However, this practice is not mandatory in the Philippines. Nine of 17 countries selected have legislation declaring TB as a notifiable disease. These countries include Australia, Canada, China, Kiribati, New Zealand, the Republic of Korea, Singapore, the United Kingdom, and Vanuatu. The other 8 countries have notification regulations in their domestic laws, but which do not specify the diseases, including TB. Examples of legislation in different countries concerning the notification of TB are presented in Table 1. Because the sample countries are mainly drawn from the Western Pacific region, our study findings are primarily applicable to countries within this region. However, given that TB is a notifiable disease in other countries such as the United Kingdom and Canada, the results of the study provide useful information in understanding international practice more broadly.

The goals of TB control are to reduce mortality, morbidity, and disease transmission until it no longer poses a threat to public health. One of the target goals of TB control is case detection. In one US study, reporting of TB cases contributed to the identification of more TB cases, which led to improved TB control. Reporting has been defined as the “obligation of health care institutions, laboratories, and health care or allied health professionals who diagnose, treat, or care for TB patients to report confirmed or suspected cases to the appropriate health agencies (CDC, 2009).” In some cases, this obligation includes notifying the appropriate agencies or authorities of the patient’s adherence or non-adherence to treatment. In the US, TB control laws have established reporting requirements even at the sub-national jurisdiction level in at least 25 different jurisdictions, based on legal research, review, and feedback provided by legal and TB practitioners [4]. Health care providers, laboratories, or others are legally required to report suspected or confirmed cases of TB, MDR-TB, or XDR-TB infection, and public health officials in state or non-state agencies are legally required to further report such information to any other entity at the appropriate local, state, or tribal levels.

Since 2012, TB has been a notifiable disease in India, which means that all cases of TB diagnosed by any means must be reported to the public health authorities following a specified format [1]. This initiative was implemented to estimate the number of TB cases in the community with greater accuracy [14]. China, a country with the second highest number of TB cases, has improved its TB estimates drastically since implementing a web-based system of mandatory case reporting in 2005 [7, 8, 24]. Other countries reviewed in this study have also enacted or revised laws concerning TB and notification requirements; thus, these countries have a current regulatory reporting system.

There is a wide-ranging consensus among many countries concerning the notification of TB. It is clear that public health legislation can facilitate more effective interventions in identifying and controlling communicable diseases. Specific public health regulations requiring the notification of TB appear to be a standard best practice norm internationally. It is likely that the Philippines would benefit from adding TB to its current list of notifiable diseases.

## Discussion

Although specified as a notifiable disease under the Law on Reporting of Communicable Diseases (Republic Act 3573) in 1929, TB was omitted from the official list of notifiable diseases because it was no longer considered to be a public health problem. However, with the increasing recognition of the continued burden of TB in the Philippines and the threat of MDR-TB, there is a need to strengthen the surveillance system as articulated and recommended in various reports. Reinstatement of TB on the list of notifiable diseases is highly recommended, and the current legislation could be amended in two ways to achieve this.

First, the Revised List of Notifiable Disease, Syndromes, Health-related Events and Conditions of 2008 could be amended to include TB. To justify this amendment, relevant information concerning the need and significance of TB notification needs to be readily available. Such information, as this study has shown, can be obtained through viewing the policies and processes of a range of developed and developing countries in this regard. In terms of TB notification, it is clear that the Philippines needs to bring its approach into line with international standards, to ensure effective TB control. Any revision should take into account the following questions: What is the definition of the notifiable disease? What kind of communicable diseases are included on the list of notifiable diseases? Who has the responsibility for reporting/notification? What is the timeframe or timeline of reporting/notification and what is the process and content of reporting/notification?

Second, the current legislation could be amended to include requirements for the identification and management of TB cases and safeguarding of individual rights; thereby, making the laws more comprehensive. The laws should detail requirements concerning: 1) the identification of TB cases (screening, examination and testing, and reporting); 2) the management of TB cases (investigations, treatments, and specific measures, such as emergency detection, quarantine, isolation, and activity-related enforcement); 3) the safeguarding of individual rights (due process, confidentiality, privacy, anti-discrimination, and religious exemptions); 4) vulnerable populations, such as those in correctional facilities and; 5) additional TB provisions, as may be necessary.

## Conclusions

A review of international standards suggests that specific legislation or a regulatory provision for ensuring that TB is notified is necessary as part of domestic health legislation. Most countries, even the smaller countries in the Western Pacific region, have a legislative basis concerning regulations on notifiable diseases, including TB, unlike the Philippines. Therefore, there is a need for revised and updated legislation concerning notifiable communicable diseases in the Philippines. Furthermore, it is strongly recommended that TB be reinstated on the notifiable diseases list in the Philippines, which can be achieved in two ways, namely, either through revising the current official list to include TB or through amending existing legislation or introducing new legislation to control TB. Any new requirements should specify methods of TB identification and management, and how individual rights are to be safeguarded. As with other countries, the Philippines could modify TB requirements either through legislation or regulation.

## Abbreviations

APSED: Asia Pacific Strategy for Emerging Diseases
IDHL: International Digest of Health Legislation
IHR: International Health Regulations
TB: Tuberculosis
